# Supplementary material for: Perceptions of barriers and facilitators for cervical cancer screening from women and healthcare workers in Ghana: Applying the Dynamic Sustainability Framework
Source: PLOS Glob Public Health. 2025 Apr 30;5(4):e0003011. doi: 10.1371/journal.pgph.0003011 (PMC12043171; doi:10.1371/journal.pgph.0003011)
Supplement: Attachment — Submitted filename: RESPONSE TO REVIEWERS .pdf [file pgph.0003011.s001.pdf]

21st September 2024

The Academic Editor
PLOS Global Public Health

RE: INVITATION TO REVISE MANUSCRIPT

Manuscript: Number: PGPH-D-24-00360

Title: Perceptions of barriers and facilitators for cervical cancer screening from women and healthcare workers in Ghana: Applying the Dynamic Sustainability Framework.

Dear Dr. Suma Nair

We express our appreciation to the you and the reviewers for the constructive critique of our manuscript. The authors agree with reviewers on many of the concerns raised in the attached manuscript. We believe the manuscript is much improved as a result and are grateful for the thoughtful and thorough review.

We have provided a point-by-point response to each reviewer comment as detailed in the attached document. Also, where substantive changes, deletions and insertion of key sentences have been made to the revised manuscripts, exact changes and where (pages/sections) effected, have been clearly indicated as part of the responses to comments.

In addition to this cover letter, we have provided a clean copy highlighting in blue font all changes made to the revised manuscript as required. A clean manuscript that includes these changes without any annotations, highlighting or comments has also been added as required. Please do not hesitate to contact me if you have further questions or comments about this manuscript.

Thank you,

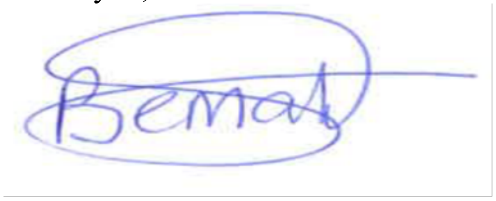

ADWOA BEMAH BOAMAH MENSAH, PhD
CORRESPONDING AUTHOR

REVIEWERS' COMMENTS AND RESPONSE

Reviewer #1: The manuscript would benefit from expanded recommendations for policy and practice, particularly detailing the operationalization of suggested interventions like self-collected HPV tests within the Ghanaian healthcare system. Addressing the limitations more comprehensively, particularly regarding sampling strategy and potential biases, along with a clearer outline of future research directions, would strengthen the manuscript. Minor editorial revisions for clarity and coherence are recommended to polish the presentation, please refer to the comments in the PDF. Addressing these areas can amplify the study's contribution to public health efforts in combating cervical cancer in low-resource settings.

Response: Thank you for the comment. We have now included a section on strengths and limitations. We have also acknowledged the potential biases and outline future studies: ". While we tried to minimize the risk of selection bias by conducting a detailed eligibility screening, this is a concern given that we relied on administrators at the healthcare facilities to assist with purposeful identification of potential healthcare worker participants. Another limitation is that we administered the survey to women visiting the CERVICARE centers, as a result we may not capture the barriers faced by women who do not visit these facilities. The time component of the Dynamic Sustainability Framework (DSF) was not factored into this cross-sectional study. As such, our study does not capture how changes over time may have influenced the barriers observed. Future studies which capture changes in the clinics over time are needed to effectively measure changes over time as detailed in the time component of the DSF." Please refer to strengths and limitations on pages 25 to 26 of the revised manuscript.

Major revision

2. Comments to the Authors

2.1 Overview

Congratulations to the authors for conducting this study on understanding barriers and facilitators to cervical cancer screening for women in Ghana. Certainly, in low- and middle-income countries and, especially those of sub-Sahara Africa, notwithstanding issues of patient and social factors such as education and health literacy, gender and culture, limited availability of screening technologies and facilities serves to compound rates of uptake. This manuscript reflects an important and prevalent situation in many low- and middle-income countries and will be of interest to the readership of PLOS Global Public Health.

Response: Thank you for taking time to review and provide us with feedback.

2.2 Major comments

The authors state that the study is designed in line with an exploratory, mixed-methods sequential approach. While I understand the author's intentions to conduct a study of this nature, there are two overarching comments I would like to make and these center on the author's use of the Dynamic Sustainability Framework (DSF) in this study and the conceptualization of the way in which this study is presented in this manuscript. The explication by the authors of the application of the DSF to this study is very lacking, indeed, I would argue, almost absent in this manuscript. At a minimum, the reporting of the study is confusing and unclear. As such, there are areas in the paper that warrant additional explanation, particularly parts of the research process and, of course, the conceptual framework of the DSF. I explain this in my following comments.

Response: Thank you for the comment. We now provide clarity about the DSF and its use in the study on pages 5-6 of the revised manuscript. Additionally, the presentation of the results has been revised per the reviewer's suggestions (pages 11 to 22).

2.2.1 Dynamic Sustainability Framework

The authors have used the DSF in this study as a conceptual framework. They have taken components of the model to inform the study design and drive the data collection process. Whilst this is not incorrect, it is completely unclear to the reader how the authors have arrived at any decision making on this use of the DSF in this study. I suspect this is why the authors state that they "…used the general DSF in combination with a mixed methods approach…". I argue this is fundamentally incorrect. The DSF is the conceptual framework of this study and should be applied and explained in this manner. To this end, what do the authors mean by "the general DSF"? Is this different from the DSF reported in various literature?

Response: Thank you for the comment. We agree with the reviewer and have revised our manuscript accordingly. The DSF is described more fully, including why it was adapted and how it was applied in this study. Please refer to the 'Conceptual Framework' section on pages 5-6 of the revised manuscript.

Moreover, in addition to unclarity as to what is the "general DSF", the authors use "ecological system" to guide the survey work and "practice setting" to guide the qualitative work. How or why was it decided to "break up" up the DSF model in this way? What was the decision making for seemingly "plucking out" these domains of the model? Is it because existing screening technologies and facilities in Ghana are considered to already fit within the "Intervention" domain and therefore the focus of this study is to unpack issues in the "practice setting/context" and "ecological system"? I think a large part of my confusion around the authors use of the DSF in this study is that Figure 1 is missing the critical detail of "practice setting". Ensuring Figure 1 is accurate will go a long way to helping readers understand this better.

Response: Thank you for this significant comment. We agree with the reviewer and have now revised the manuscript to help readers better understand our approach. We considered the existing

cervical cancer screening program in Ghana as the intervention as it fits within the intervention domain of the DSF. Hence this study focused on the issues in the practice setting and ecological system to generate policy and intervention driven data that will potentially improve the uptake of the screening services by women. Please refer to the last paragraph under "introduction" on page 5 and section on 'Conceptual Framework' on pages 5-6 of the revised manuscript for relevant details. We no longer break up the model but rather consolidate it accordingly. A revised figure has also been added to the submission (refer to Figure 1, supplementary file).

2.2.2. Reporting

In view of the confusion that this paper generates for the reader, particularly regarding the use of the DSF, it is my suggestion that the authors consider adding in a new section named "Conceptual Framework" and spending time to more deeply conceptualize the use of the DSF to this study. Further, the DSF in a new section can be better explained, including what the domains are all about and how they apply to this type and focus of study, for example, to the uninitiated reader, there is no explanation of what "ecological system" theoretically means. In so doing, the authors can then give time to explaining how such constructs informed the approach to data collection. Currently, there is no reference to the application of the constructs to the data collection in either the quant or qual study.

Response: Thank you for this significant comment. We have now created a section titled "Conceptual Framework" to discuss the DSF and its domains as well as operationalize how it is applied in our study. Please refer to pages 5-6 of the revised manuscript. The methods section has also been modified to include information on the types of questions used in both the quantitative study and qualitative study pertain to various domains of the DSF. Please refer to pages 9 to 10 for the quantitative study and page 18 for the qualitative study.

On the point of reporting, this is a challenging paper to read. There are sections of the research process, for example, eligibility (line 186), recruitment (sampling approach) (line189) and consent (line 193) are reported in the data collection section. Data collection should only focus on the instrument used. Another example of the challenge that this paper presents to the reader is the reporting of results of the quant survey after the qualitative data collection section – this makes it difficult to decipher how the findings of the quant survey were developed to inform the qual data collection/interview guide. It is my suggestion that the authors might like to reconsider restructuring the reporting of the study as two distinct studies, to enhance clarity throughout. For instance, study 1 is the quant study and would align with standard reporting criteria – design and setting, sample and sampling, recruitment and consent, data collection, data analysis, results. The introduction of study 2 will therefore be better positioned, clearer and logically placed in the paper and can be reported similarly – design and setting, sample and sampling, recruitment and consent, data collection, data analysis, results. A discussion section can then follow that discusses the overall findings and, important, is discussed in view of the principles of the DSF – which, currently, is lacking in the discussion. Such an approach to restructuring the paper would also help

prevent what appears to be a randomly placed section in the paper on “Ethics approval and consent process”, when the consent processes have already been previously discussed (albeit incorrectly) in the quant and qual data collection sections.

Response: We have now restructured the methods section as suggested by the reviewer. Each sub-section has been reformatted to contain information only pertaining to the specific sub-section. Additionally, more clarity has been provided to separate the quantitative and qualitative portions of the research. Study I highlight the survey methods and corresponding results while study II presents the qualitative methods and results. Please refer to pages 7-22 of the revised manuscript.

2.3 General comments

2.3.1 Quant survey study

Line 148 states that “individuals were selected” to participate. Is this correct? How exactly were they recruited? Was there a decision-making process on which women were approached? Is it possible that the ‘participant information and consent sheet’ used in this study could be included as supplementary material?

Response: Thank you for the comment. We have now described the recruitment and consent process under a sub-section on “recruitment and consent” under Study I (page 9) and study II (pages 17-18) of the revised manuscript. Participant information sheet and consent form used have been included as supplementary materials.

Was a sample size calculated for this first phase of the study? There is no mention of this in the paper.

Response: Thank you for the comment. We have now included this detail. It reads: “We estimated the sample size using the formula: n=(N−1)·e2+Z2·p·(1−p)N·Z2·p·(1−p) where population size (N) is 321 (based on 2021 attendee number from facilities), the Z-value (Z) for a 95% confidence level is 1.96, the population proportion (p) is conservatively estimated at 0.5, and the margin of error (e) is set at 0.05. We then calculated an attrition rate of 20%, bringing the estimated sample size to 210. However, at the end of the study, we had surveyed 215 women.” Please refer to page 8 of the revised manuscript.

The issue of bias is not addressed – and, on this note, a strengths and limitations section are not included in the paper. Bias might have been an issue as women were recruited from centres. This assumes only those women able to attend centres were included in the study. Moreover, the issue of payment made to participants is not addressed as a potentially biasing factor.

Response: Thank you for drawing our attention. We have now included a section on strengths and limitations acknowledging all potential biases on pages 25-26 of the revised manuscript.

More information is needed on the tool used, for example, how many factors and items did it originally start with? Was it a validated tool in the Ugandan study? What part of the original tool was adapted? After adaption, was face and content validity tested before dissemination to the participants?

Response: Thank you for this comment and raising the issue of the validity of the instrument. We have revised the methods to include more information about the quantitative survey within the data collection sub-section (pages 9-10 of revised manuscript). The survey was adapted from an instrument used for a similar study in Uganda. Their survey was pre-tested prior to use. Our survey was assessed for clarity, but we did not directly examine the content and face validity of the tool. We have included this in the strengths and limitations section on pages 25-26 of the revised manuscript.

2.3.2 From quantitative-to-quantitative methods

How was the importance of quantitative findings decided over others in terms of informing what would be asked in the interviews?

Response: We interpreted this comment in terms of how the survey findings influenced the interviews, particularly the questions asked. Assuming this is the correct interpretation, we now indicate under the "data collection and procedure" section of study II, on page 18 of the revised manuscript that key findings from the quantitative data (such as long wait time at the clinic, distance to health facilities etc.) were related to the practice setting and ecological system of the DSF. This influenced the interview questions that were asked of healthcare workers. We also discuss how the use of healthcare workers interviews provides a deeper context of the practice settings domain of the DSF because healthcare workers have intimate knowledge of the practice setting.

Why was it decided to only interview health workers perceptions on health system challenges experienced by women? Having not interviewed women themselves seems to be a loss in this study.

Response: Thank you for the comment. We interviewed HCWs in the qualitative phase following the interviews with women and based on their responses. We gained insight on the barriers faced by women based on the survey that was administered to them as detailed in the paper. Our goal in also interviewing the HCWs was to corroborate what women were reporting and to determine whether there were other concerns that weren't captured. We have however acknowledged this as a limitation on pages 25-26 of the revised manuscript.

Quantitative findings are reported in this short section before the formal Results section.

Response: Thank you for noting this error. We have moved these findings to the Results section, on pages 11 to 16 of the revised manuscript.

2.3.3 Qualitative study

The authors state that the administrators identified potential participants – based on what criteria? Bias in this process will need to be addressed.

Response: Thank you for raising this concern. The administrators served as "recruitment links" for the healthcare worker participants only in Study II. The sampling approach in Study II was purposive, hence we relied on the hospital administrators to link the trained research assistants (RA) to healthcare workers who were involved in cervical cancer screening in each study facility. The research assistant subsequently met the potential participants to determine their eligibility for inclusion in the study, and if eligible, they were invited to participate. We now detail the recruitment process on pages 17-18 of the revised manuscript, under the "Recruitment and consent" section. We also acknowledge this approach to recruitment as a limitation on pages 25-26 of the revised manuscript.

Results of the qual study (e.g., sample size of 17) is reported in amongst the eligibility, recruitment and consent information (which supports my earlier point about restructuring this paper to report it in line with standard reporting criteria). Data analysis is also reported in this section, for example, information about how theoretical saturation was arrived at should be in a dedicated data analysis section.

Response: Thank you. We have restructured Study II as suggested (please refer to pages 16 – 22 of the revised manuscript). We have also adjusted the sub headings to clearly separate recruitment practices, the data collection process, and the results of the qualitative interviews.

2.3.4 Results

Line 329 – "based on the results of the quantitative analysis, two main themes related to the practice setting were further explored…." – how were these themes arrived at from the quant data to know that they should be explored in the interviews?? Was there additional theoretically driven analysis that occurred with the quant data, other than the descriptive and analytical statistical approaches stated?

Response: Thank you for the comment. That was an error. This correction has been made on page 20 of the revised manuscript.

The End.
