# Supplementary material for: Perceptions of barriers and facilitators for cervical cancer screening from women and healthcare workers in Ghana: Applying the Dynamic Sustainability Framework
Source: PLOS Glob Public Health. 2025 Apr 30;5(4):e0003011. doi: 10.1371/journal.pgph.0003011 (PMC12043171; doi:10.1371/journal.pgph.0003011)
Supplement: Attachment — Submitted filename: Cervical-screening_Ghana_20 Dec 2024.pdf [file pgph.0003011.s002.pdf]

Perceptions of barriers and facilitators for cervical cancer screening from women and healthcare workers in Ghana: Applying the Dynamic Sustainability Framework.

Manuscript reference number: PGPH-D-24-00360R1

Revised submission – research paper

Recommendation

Major revision

Comments to the Authors

Conceptual Framework

The new content makes a valuable addition to the paper. This makes it clear to the reader not only the construction of the DSF but how the authors have interpreted it and applied it in their study. This section demonstrates their understanding of some of the key principles of the DSF, for example, constant change in an intervention and how such change serves to influence the ongoing success or otherwise of the intervention.

My main critique of this section, however, is that the authors have, in the main, overlooked the integration of the tenets of the DFS model into their research. The tenets (or principles) of a model are what researchers take from a model to use in their research, either to guide development, implementation and evaluation or to test those tenets with the view of developing the theory in the given field. I wonder if the authors have somewhat done this, that is, applied the tenets of the DSF to the development of the materials in their research as they mention in lines 154-155 that, “With this understanding, we developed our study tools”. Conveying this in this section of the paper would complete this section to a standard required of demonstrating theoretical competency. As it currently reads, this is “brushed over”, which makes the reader, in turn, wonder if the authors deeply understand the application of theory in research.

Line 119: It is, not “it’s”.

Quantitative survey

Design and setting

Line 174: The authors state that they have adopted a cross-sectional design to investigate… but this sentence should finish with stating ‘in what population’ and ‘where’ (i.e., …in adult women aged 30 years and over in Ghana.).

Line 176: “allowing us to analyze the status of cervical cancer screening barriers” – this statement appears to contrast with the first sentence in this paragraph, that the authors have conducted the survey to investigate the barriers and facilitators of cervical cancer screening. Moreover, the terminology, “the status of cervical cancer screening barriers…” is an unusual way use of wording. Please consider rewording.

Sample and sampling

Line 196: "…at the end of the study, we had surveyed 215 women". Please move this to the Result section.

Recruitment and consent
Line 208: The authors mention they had an "ethical approval letter" – which university provided the ethical approval?

Line 213: Please change "research assistances" to research assistants.

Data collection and procedure
My major critique of this section relates to the author's understanding of the constructs of the DSF and how such constructs (and the tenets that underpin the constructs). In Lines 241-245, the authors state that the primary purpose of the survey is to understand 'something' about Ghanian women eligible for screening (this isn't clear), but then state that this pertains to the ecological systems domain of the DSF. The ecological systems construct of the DSF, whilst it includes 'Other practice settings', in the main it includes macro-level considerations such as policy, regulations, market considerations and population level considerations. The authors describe their data collection tool as collecting information on participant demographics, knowledge of cervical cancer and HPV vaccination, symptoms, knowledge and belief scales, practice setting considerations, and sexual history. The links to or relationship as to how these domains of the data collection tool support the DSF ecological systems construct is not clear and, moreover, I would argue, incorrectly conceptualised.

Line 223: Please change "assess" to assessed.

Lines 243-245: Again, the authors talk here only of wanting to ascertain the barriers to screening. Consistency should be reviewed throughout the manuscript to ensure that identification of any facilitators are also included as a goal of this study.

Statistical analysis
The 'fourth section' of the data collection tool doesn't appear to have an analytical approach stated.

What approach to analysis was conducted for the Likert scale data, the 'fifth section' of the tool?

Results
The knowledge and belief scales do not appear to be reported.

Qualitative study
Design and setting
What is research question for the qualitative study?? What did the interview process actually aim to generate in terms of new knowledge?

Lines 308-311: The authors state the key findings of the quant study informed what the interview scheduled asked. It would be immensely valuable to this manuscript if the

authors provided a succinct statement on what those key findings were that informed the interview schedule. It is one thing to report the results descriptively in the previous section but then another to state in the introduction to the qual study that “key findings” informed the interviews.

Lines 314-315: The authors state that an “exploratory sequential research design was employed, integrating quantitative and qualitative methods…”. To my knowledge, there are only qualitative data involved in this stage of the research. If the authors intention here is to describe the overall study design then this statement needs to be moved further up the manuscript to enable the reader clarity to understand what design this research comprises.

Sample and sampling procedure

Most of the content in this sub-section needs to be moved to the Data analysis section as it discusses analysis.

Recruitment and consent

Line 324: Regarding the term, “gatekeeper”, do the authors mean stakeholder?

Data analysis

Is there a reference that the authors can cite to demonstrate which or what type of “inductive thematic analysis framework” was used?

Discussion

My main critique with the content in the Discussion is the claim that the findings discussed, or some of the findings, “…pertain to the ecological systems domain…”. In what ways do the data that centre on ‘lack of knowledge’ respond to the construct of ecological systems? Lack of knowledge is arguably a micro-level, intrapersonal factor but I appreciate could be influenced by meso-level, interactional factors and/or macro-level, institutional factors. If this is what the authors were aiming to convey (i.e., this multi-level complexity on behaviour, health seeking and risk reducing practices), then this needs to be much more clearly explicated and argued.

The remainder of the Discussion section does little to develop an argument that explicates how the findings are informed by, apply to or develop the constructs of ‘practice setting’ and or ‘ecological systems’.

Strengths and limitations

The modified DSF?? What did the authors do to modify or adapt the DSF?? I appreciate that the authors have changed the graphic or schematic of the model but what were the conceptual or theoretical implications of this to the DSF??
