# Supplementary material for: Perceptions of barriers and facilitators for cervical cancer screening from women and healthcare workers in Ghana: Applying the Dynamic Sustainability Framework
Source: PLOS Glob Public Health. 2025 Apr 30;5(4):e0003011. doi: 10.1371/journal.pgph.0003011 (PMC12043171; doi:10.1371/journal.pgph.0003011)
Supplement: Attachment — Submitted filename: RESPONSE TO REVIEWER COMMENTS.pdf [file pgph.0003011.s003.pdf]

3rd April 2, 2025

The Academic Editor
PLOS Global Public Health

RE: INVITATION TO REVISE MANUSCRIPT

Manuscript Number: PGPH-D-24-00360R1
Revised Submission: Research Paper

Title: Perceptions of barriers and facilitators for cervical cancer screening from women and healthcare workers in Ghana: Applying the Dynamic Sustainability Framework.

Dear Dr. Suma Nair

We thank you and the reviewers for the constructive critique of our manuscript. The authors agree with many of the concerns raised and have addressed them in the attached manuscript. We believe the manuscript is much improved as a result and are grateful for the thoughtful and thorough review.

We have provided a point-by-point response to each reviewer's comment, as detailed in the attached document. Also, where substantive changes, deletions, and insertions of key sentences have been made, exact changes and location (pages/sections) are indicated as part of the responses to comments.

In addition to this cover letter, we have provided a clean copy highlighting in yellow all changes made to the revised manuscript as required. A clean, revised manuscript without any annotations, highlighting, or comments has also been added as required. Please do not hesitate to contact me if you have further questions or comments about this manuscript.

Thank you,

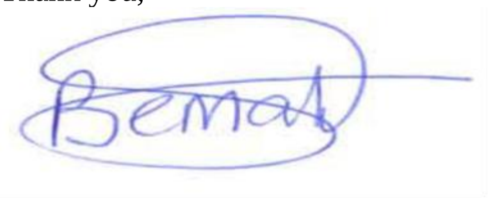

ADWOA BEMAH BOAMAH MENSAH, PhD
CORRESPONDING AUTHOR

REVIEWERS' COMMENTS AND RESPONSE

Comments to the Authors

1. **Conceptual Framework**

The new content makes a valuable addition to the paper. This makes it clear to the reader not only the construction of the DSF but also how the authors have interpreted it and applied it in their study. This section demonstrates their understanding of some of the key principles of the DSF, for example, constant change in an intervention and how such change serves to influence the ongoing success or otherwise of the intervention. My main critique of this section, however, is that the authors have, in the main, overlooked the integration of the tenets of the DFS model into their research. The tenets (or principles) of a model are what researchers take from a model to use in their research, either to guide development, implementation, and evaluation or to test those tenets with the view of developing the theory in the given field. I wonder if the authors have somewhat done this, that is, applied the tenets of the DSF to the development of the materials in their research as they mention in lines 154-155, "With this understanding, we developed our study tools". Conveying this in this section of the paper would complete this section to a standard required of demonstrating theoretical competency. As it currently reads, this is "brushed over", which makes the reader, in turn, wonder if the authors deeply understand the application of theory in research.

Response: Thank you for the comment. As suggested by the reviewer, we have now revised the description of the conceptual frame to reflect how it was adapted in the current study. (refer to "Conceptual framework on pages 5-6 of the revised manuscript). We have further described the study tools for Study I and Study II, informed by the practice setting and ecological system domains of the DSF. Please refer to page 9, lines 225 to 231 of the revised manuscript.

2. Line 119: It is, not "it's".

Response: We have deleted the paragraph that includes this phrase to reduce redundancy.

3. **Quantitative survey**

Design and setting

Line 174: The authors state that they have adopted a cross-sectional design to investigate… but this sentence should finish with stating 'in what population' and 'where' (i.e., …in adult women aged 30 years and over in Ghana

Response: Thank you for the comment. We have revised the sentence per the reviewer's suggestion so that it now reads as follows: "We used a cross-sectional design to investigate the barriers and

facilitators of cervical cancer screening in women aged 30 years and over in Ghana." Please refer to page 7, lines 171 to 172.

Line 176: "Allowing us to analyze the status of cervical cancer screening barriers" – this statement appears to contrast with the first sentence in this paragraph, which the authors have surveyed to investigate the barriers and facilitators of cervical cancer screening. Moreover, the terminology, "the status of cervical cancer screening barriers…" is an unusual way use of wording. Please consider rewording

Response: Thank you for the comment. We have deleted the paragraph that includes this phrase to reduce confusion.

Sample and sampling

Line 196: "…at the end of the study, we had surveyed 215 women". Please move this to the Result section.

Response: Thank you for the suggestion. We have now moved the sentence to the Result section. Refer to page 11, line 256 of the revised manuscript. The sentence reads, "A total of 215 women were surveyed."

Recruitment and consent

Line 208: The authors mention they had an "ethical approval letter" – which university provided the ethical approval?

Response: Thank you for the comment. We have now included the Universities that provided the ethical approval: Committee on Human Research, Publication and Ethics, Kwame Nkrumah University of Science and Technology, Ghana (CHRPE/AP/043/22) and the Cancer Center Protocol Review Committee, University of Minnesota, USA (CPRC# 2022LS045). Please refer to page 8, lines 204 to 207 (Study I), and pages 16 to 17, lines 309 to 311 (Study II) of the revised manuscript.

Line 213: Please change "research assistances" to research assistants.

Response: Thank you for the comment. "Research assistants" has been changed to "research assistants." Refer to page 9, line 211.

Data collection and procedure

My major critique of this section relates to the author's understanding of the constructs of the DSF and such constructs (and the tenets that underpin the constructs). In Lines 241-245, the authors state that the primary purpose of the survey is to understand 'something' about Ghanaian women eligible for screening (this isn't clear), but then state that this pertains to the ecological systems domain of the DSF. The ecological systems construct of the DSF, whilst it includes 'Other practice

settings', in the main it includes macro-level considerations such as policy, regulations, market considerations, and population level considerations. The authors describe their data collection tool as collecting information on participant demographics, knowledge of cervical cancer and HPV vaccination, symptoms, knowledge and belief scales, practice setting considerations, and sexual history. The links to or relationship as to how these domains of the data collection tool support the DSF ecological systems construct is not clear and I would argue, incorrectly conceptualized.

Response: The Authors agree with the reviewer's critique. This study is part of a larger study to optimize HPV-based cervical cancer screening in Ghana. This manuscript reports on barriers and facilitators to existing cervical cancer screening services. We have, therefore, revised the manuscript to reflect this aspect of data collection and have described how the revised domains of the data collection tool support the DSF constructs. All other unrelated sections of the data collection tool have been deleted. These changes are reflected on page 9, lines 222 to 231.

Line 223: Please change "assess" to assessed.

Response: Thank you for the comment. We have changed "assess" to "assessed" (Line 221, page 9).

Lines 243-245: Again, the authors talk here only of wanting to ascertain the barriers to screening. Consistency should be reviewed throughout the manuscript to ensure that identification of any facilitators is also included as a goal of this study.

Response: Thank you for the comment. We have reviewed the manuscript and revised it as needed for consistency. " Facilitator" has been deleted accordingly. These changes are noted throughout the manuscript. For example, on line 53, page 2; line 119, page 4; line 152, page 6; line 171, page 7; and line 221, page 9.

Statistical analysis

The 'fourth section' of the data collection tool doesn't appear to have an analytical approach stated.

What approach to analysis was conducted for the Likert scale data, the 'fifth section' of the tool?

Response: Thank you for the comment. In response to an earlier reviewer comment, we have revised the manuscript to focus on the sections of the survey that had questions to identify potential barriers and facilitators to screening uptake that reflect the practice setting and ecological system domains of the DSF. We have now described the analytical approach for the two relevant sections of the data collection tool. Analytical approaches for the other sections of the data collection tool were deleted to be responsive to the reviewer. (Please see "data collection tools and procedure" on page 9),

Results

The knowledge and belief scales do not appear to be reported.

Response: Thank you for this comment. As noted above, the primary purpose of this manuscript is to inform our understanding of potential barriers and facilitators to screening uptake based on responses to questions that pertain to the practice setting and ecological system domains of the DSF. We have deleted the knowledge and belief scales to be responsive to an earlier comment from the reviewer. (Please see "data collection tools and procedures" on page 9).

4. **Qualitative study**

Design and setting

What is the research question for the qualitative study? What did the interview process aim to generate in terms of new knowledge?

Response: We now include a research question for the qualitative study, which reads as follows: "What are the potential barriers and facilitators that women encounter when accessing cervical cancer screening services at a Ghana Health Services (GHS) CERVICARE center?" Please refer to page 16, lines 298 to 300, page 16 of the revised manuscript.

Lines 308-311: The authors state the key findings of the quantitative study informed what the interview schedule asked. It would be immensely valuable to this manuscript if the authors provided a succinct statement on what those key findings were that informed the interview schedule. It is one thing to report the results descriptively in the previous section but another to state in the introduction to the qualitative study that "key findings" informed the interviews.

Response: We now note where these are described in detail to avoid redundancy. *"Key findings from the quantitative data (such as long wait time at the clinic, and distance to health facilities) related to the practice setting domain of the DSF influenced which interview questions were asked to healthcare workers".* We have also now provided key findings that informed the interview schedule (refer to lines 328 to 332, page 17 of the revised manuscript).

Lines 314-315: The authors state that an "exploratory sequential research design was employed, integrating quantitative and qualitative methods…". To my knowledge, there are only qualitative data involved in this stage of the research. If the authors' intention here is to describe the overall study design, then this statement needs to be moved further up the manuscript to enable the reader clarity to understand what design this research comprises.

Response: Thank you for the comment. We have deleted the sentence that includes this phrase. Refer to "Study design and setting" on page 16 of the revised manuscript.

Sample and sampling procedure.

Most of the content in this subsection needs to be moved to the Data analysis section as it discusses analysis.

Response: Thank you for the comment. The heading of this sub-section has now been revised (Sampling procedure and sample size determination) to reflect the content. The authors described how sample size is determined in a qualitative study with a citation. Please refer to page 16, lines 301 to 306 of the revised manuscript.

Recruitment and consent

Line 324: Regarding the term, “gatekeeper”, do the authors mean stakeholder?

Response: Thank you for the comment. The sentence has been revised as follows: “Approval for this study was provided by the ……” Please refer to page 16, line 308 of the revised manuscript.

Data analysis

Is there a reference that the authors can cite to demonstrate which or what type of “inductive thematic analysis framework” was used?

Response: Thank you for the comment. We have extensively revised this section and now include relevant references, which are as follows: (1) Miles MB. Qualitative data analysis: An expanded sourcebook. Thousand Oaks. 1994 (Refer to page 18, line 342); (2) Chambers DA, Glasgow RE, Stange KC. The dynamic sustainability framework: addressing the paradox of sustainment amid ongoing change. Implement Sci. 2013 Dec;8(1):117. (Refer to pages 18 to 19, lines 336 and 357).

5. Discussion

My main critique of the content in the Discussion is the claim that the findings discussed or some of the findings, “…pertain to the ecological systems domain…”. In what ways do the data that centre on ‘lack of knowledge’ respond to the construct of ecological systems? Lack of knowledge is arguably a micro-level, intrapersonal factor but I appreciate could be influenced by meso-level, interactional factors and/or macrolevel, institutional factors. If this is what the authors were aiming to convey (i.e., this multi-level complexity on behaviour, health seeking and risk reducing practices), then this needs to be much more clearly explicated and argued.

The remainder of the Discussion section does little to develop an argument that explicates how the findings are informed by, apply to or develop the constructs of ‘practice setting’ and or ‘ecological systems.

Response: Thank you for the comment. The discussion has been revised to reflect how the practice setting and ecological system of the DSF apply to the findings. Please refer to the sub-section “Discussion” on pages 22 to 25 of the revised manuscript.

6. Strengths and limitations

The modified DSF?? What did the authors do to modify or adapt the DSF?? I appreciate that the authors have changed the graphic or schematic of the model, but what were the conceptual or theoretical implications of this to the DSF??

Response: The term “modified” was used because of the changes authors made to the graphic or schematic of the DSF model. However, this graphical change has no conceptual or theoretical implications for the DSF model. To avoid any potential miscommunication, the term “modified” has been deleted. Please refer to page 25, line 497 of the revised manuscript. We have also added a limitation related to the DSF on page 26, lines 511 to 513.

The End
